# 82032 Lessons learned from a virtual engagement salon amidst a pandemic

**DOI:** 10.1017/cts.2021.624

**Published:** 2021-03-31

**Authors:** Pierre Khalil, Jordan Louis, Barbara Tafuto, Marsha Gordon

**Affiliations:** Rutgers University

## Abstract

ABSTRACT IMPACT: This work is intended to improve community engagement salons both virtually and generally in order to maximize the benefit of this vital research tool. OBJECTIVES/GOALS: The Meharry-Vanderbilt Community Engaged Research Core (CERC) provides a protocol to maintain high standards in community engagement studios, even in a virtual setting amidst a pandemic. A virtual community engagement salon was selected as a case study to evaluate outcomes. METHODS/STUDY POPULATION: A virtual salon regarding sun safety in the Latinx community was observed live via Zoom and as a recording afterward. Following dissemination and completion of the post-meeting surveys, authors compiled and reviewed the results. An assessment was developed to determine the salon’s alignment with the Meharry-Vanderbilt CERC guidelines in a virtual setting; this was designated as the primary outcome. Data from the session were compared to the available literature on the topic, which produced three subheadings to the secondary outcome essential to the success of virtual community engagement salons: researcher preparedness, participant selection, and survey importance. RESULTS/ANTICIPATED RESULTS: The CERC guidelines of the community engagement salon were met and were effectively translated into a virtual setting. The presentation given by the researcher followed all technical instructions, yet it was clear that the researcher’s demeanor and conversational soft-skills were lacking. Instead of the recommended bi-directional flow of conversation, the conversation flow shifted to a unidirectional state controlled by the participants. Following the session, only three participants completed the survey along with the researcher. This completion rate of under 50% provides limited feedback on participants’ perspectives on the session’s quality and points to improve future sessions. DISCUSSION/SIGNIFICANCE OF FINDINGS: Pre-meeting researcher preparation is necessary to engage community stakeholders effectively. The lack of completed surveys from participants suggests potential fatigue from leading a majority of the conversation. Results demonstrate that solely meeting the requirements of the CERC does not suffice.

